# Supplementary material for: Two-dimensional electronic spectroscopy reveals liquid-like lineshape dynamics in CsPbI3 perovskite nanocrystals
Source: Nat Commun. 2019 Oct 31;10:4962. doi: 10.1038/s41467-019-12830-1 (PMC6823484; doi:10.1038/s41467-019-12830-1)
Supplement: Supplementary file 1 — Supplementary Information [file 41467_2019_12830_MOESM1_ESM.pdf]

Supplementary Information

Two-Dimensional Electronic Spectroscopy reveals liquid-like lineshape dynamics in $CsPbI_3$ perovskite nanocrystals

Seiler et al.

Supplementary Note 1 : Determination of average number of excitations per particle

When considering individual $CsPbI_3$ nanocrystals, we can assume that the overall number of photons absorbed per nanocrystals is small (~ 1 photon per nanocrystal). In such a case, the average number of excitations per nanocrystal ($\langle N \rangle$) is given by:

$$\langle N \rangle = \langle N_{\text{photons}} \rangle \sigma_{(E_{\text{pump}})} \quad (1)$$

Where $\langle N_{\text{photons}} \rangle$ is the photon flux (photons cm^{-2}) and $\sigma_{(E_{\text{pump}})}$ is the absorption cross-section at a given pump energy (in our case 3.1 eV). Knowing this, immediately following excitation the number of nanocrystals possessing N excitations (n_N) can be expressed in terms of Poisson statistics, such that:

$$n_N = \frac{n \langle N \rangle^N e^{-\langle N \rangle}}{N!} \quad (2)$$

where n is the total number of nanocrystals in the sample. At early times and high fluences, induced absorption processes can lead to multiexciton formation, meaning each individual nanocrystal will contribute differently to the overall photoluminescence intensity, depending on the number of photons absorbed. However, these multiexcitons decay into single excitons on a timescale of 10-200 ps in $CsPbI_3$ (*1*, *2*). Thus, after this time period, each nanocrystal will contribute equally to the overall PL intensity, such that the contribution of each individual nanocrystal to the overall intensity is given by:

$$I = 1 - I(0) = 1 - e^{-\langle N \rangle} = 1 - e^{-\langle N_{\text{photons}} \rangle \sigma_{3.1\text{eV}}} \quad (3)$$

The overall intensity at late times is therefore dependent on the total nanocrystal concentration, which is related to the PL saturation magnitude (I_0). To obtain the total PL intensity, the above expression is multiplied by I_0:

$$I = I_0 \left(1 - \mathrm{e}^{-\langle N_{\text{photons}} \rangle \sigma_{3.1\text{eV}}}\right) \quad (4)$$

The maximum PL intensity is found at t = 2 ns at multiple fluences from streak traces, similar to that shown in Supplementary Figure 3. The fit of these values to the above expression can be seen in Supplementary Figure 6, which yields an absorption cross section of 5.67×10^{-15} cm^2 for the $CsPbI_3$ nanocrystals studied here, consistent with previous values found in the literature (*3*, *4*). This results in $\langle N \rangle$ to be in the range of 0.01 – 11.5 for the fluences used in the t-PL measurements. Due to the larger absorption cross-section for these materials at higher energies (*4*), it can be concluded that the fluences used in the 2DES experiments result in $\langle N \rangle \ll 1$.

Supplementary Note 2: Extraction of the anti-diagonal linewidth from the 2D spectra

From each 2D spectrum, the anti-diagonal linewidth was extracted in the following way:

1. The coordinates of the peak maximum ($E_{1,\max}$, $E_{3,\max}$) were found (green dot on Supplementary Figure 8).
2. The main diagonal of the 2D spectrum ($E_1 = E_3$) was traced (dashed black line on Supplementary Figure 8).
3. The parallel to the main diagonal passing through ($E_{1,\max}$, $E_{3,\max}$) was found (red line on Supplementary Figure 8)
4. The perpendicular to the red line was found (blue line on Supplementary Figure 8): this is the peak' anti-diagonal spectral projection
5. From the anti-diagonal spectral projection, one can find the Full-Width at Half-maximum (FWHM).
6. The FWHM can then be extracted for each of the anti-diagonal spectral projections. These are the widths shown in Fig. 3d-f for the different samples.

Supplementary Discussion: Modelling the 2D spectra with the cumulant expansion and multimode Brownian oscillator model

In the pump-probe geometry implementation of 2DES, the sum of the rephasing and non-rephasing diagrams are emitted in the probe direction and sent to the spectrometer. Thus one measures the absorptive 2D spectrum (*5*):

$$S_{\text{Absorptive}}(\omega_1, t_2, \omega_3) = B \cdot \left[R_{\text{R}}(-\omega_1, t_2, \omega_3) + R_{\text{NR}}(\omega_1, t_2, \omega_3) \right] \tag{5}$$

Where B is a constant, R_{R} is the sum of all possible rephasing pathways and R_{NR} the sum of all possible non-rephasing pathways.

Using the cumulant expansion to second order, R_{R} and R_{NR} can be expressed as a sum of two-point correlation functions. In the case of a two-level system, the expressions are given by:

$$R_{\text{R}}\ (t_1, t_2, t_3) = \mathrm{i}\mu_{01}^4 \mathrm{e}^{-\mathrm{i}\omega_{01}(t_3 - t_1)} \mathrm{e}^{-g(t_1)+g(t_2)-g(t_3)-g(t_1+t_2)-g(t_2+t_3)+g(t_1+t_2+t_3)} \tag{6}$$

$$R_{\text{NR}}\ (t_1, t_2, t_3) = \mathrm{i}\mu_{01}^4 \mathrm{e}^{-\mathrm{i}\omega_{01}(t_3 + t_1)} \mathrm{e}^{-g(t_1)-g(t_2)-g(t_3)+g(t_1+t_2)+g(t_2+t_3)-g(t_1+t_2+t_3)} \tag{7}$$

Where ω_{01} is the transition frequency from level 0 to level 1 and μ_{01} is the transition dipole moment. The function *g(t)* is called the lineshape function,

$$g(t) = \int_0^t d\tau \int_0^\tau d\tau' C(\tau') \tag{8}$$

The correlation function *C(t)* is expressed as $\langle \delta\omega_{01}(t)\delta\omega_{01}(0) \rangle$, where $\delta\omega_{01}(t)$ is the fluctuating part of the transition frequency, as illustrated in Fig. 2d.

Thus one can model the 2D spectra with the following ingredients: 1) The average transition frequency of the states 2) the transition dipole moments and 3) the lineshape functions.

Oftentimes, a multitude of processes contribute to dephasing and hence to the lineshape function. A convenient way to include multiple contributions to the lineshape is via the **Multimode Brownian Oscillator** (MBO) model (*6*, *7*). The MBO considers that the lineshape function *g(t)* can be decomposed in a sum of contributions from multiple uncorrelated bath modes *q*:

$$g(t) = \sum_{q} g^{(q)}(t) \tag{9}$$

We shall now describe how the MBO model was employed for modelling the 2D spectra of the CdSe and $CsPbI_3$ nanocrystals.

Calculations were carried out using the code available at https://github.com/spalato/Mbo.jl .

Modelling the CdSe nanocrystals

The CdSe nanocrystals were modelled using:

$$g(t) = g^{(\mathrm{H})}(t) + g^{(\mathrm{I})}(t) + g^{(\mathrm{HR})}(t), \tag{10}$$

Where $g^{(\mathrm{H})}(t)$ represents homogeneous dephasing, $g^{(\mathrm{I})}(t)$ represents inhomogeneous dephasing and $g^{(\mathrm{HR})}(t)$ is the lineshape function for coherent oscillations (Huang-Rhys). Explicitly, the lineshape functions can be expressed as:

$$g^{(\mathrm{H})}(t) = \gamma \tag{11}$$

$$g^{(\mathrm{I})}(t) = 0.5(\sigma t)^2 \tag{12}$$

$$g^{(\mathrm{HR})}(t) = S^{(\omega_{\mathrm{vib}})}\left(\coth\left[\frac{\hbar\omega_{\mathrm{vib}}}{2k_{\mathrm{B}}T}\right]\left[1-\cos(\omega_{\mathrm{vib}}t)\right] + i\left[\sin(\omega_{\mathrm{vib}}t) - \omega_{\mathrm{vib}}t\right]\right) \tag{13}$$

Where γ is the pure dephasing rate, σ is the standard deviation of the inhomogeneous Gaussian distribution, $S^{(\omega_{\mathrm{vib}})}$ is the Huang-Rhys coupling factor, ω_{vib} is the frequency of the vibrational mode, k_{B} is the Boltzmann factor and T is the temperature.

The model parameters are summarized in Supplementary Table 2. More details about the model are also available in some recent work by our group (*8*).

Modelling the $CsPbI_3$ nanocrystals

We model the $CsPbI_3$ 2D spectra using a single effective bath mode q_{L}, which obeys the Langevin equation:

$$\ddot{q}_{\mathrm{L}} + \gamma \dot{q}_{\mathrm{L}} + \omega_{\mathrm{L}} q_{\mathrm{L}}^{2} = f(t) \tag{14}$$

Where *f(t)* is a random Langevin force and γ represents the friction (dissipative) term. In the case of a strongly overdamped oscillator, $\gamma \gg 2\omega_L$, it can be shown that the corresponding lineshape function is given by (*9*):

$$g^{(\mathrm{K})}(t) = \Delta^{2}\tau_{\mathrm{c}}^{2}\left[\frac{t}{\tau_{\mathrm{c}}} - 1 + e^{-t/\tau_{\mathrm{c}}}\right] \tag{15}$$

Where $\tau_{\mathrm{c}} \equiv \gamma / \omega_{\mathrm{L}}$ and Δ represents the amplitude of the fluctuations, which are assumed to take a Gaussian form with finite correlation time:

$$\left\langle \delta\omega(t)\delta\omega(0) \right\rangle = \Delta^{2} e^{-|t|/\tau_{\mathrm{c}}} \tag{16}$$

This overdamped oscillator model describes dissipative, irreversible processes such as solvation, collective dynamics in disordered systems and polaron formation. This functional form is often called the Kubo lineshape.

The model parameters employed to calculate the 2D spectra of $CsPbI_3$ are summarized in Supplementary Table 3. The sensitivity of the modelled lineshape behaviour to changes in the value of Δ is shown in Supplementary Figure 10.

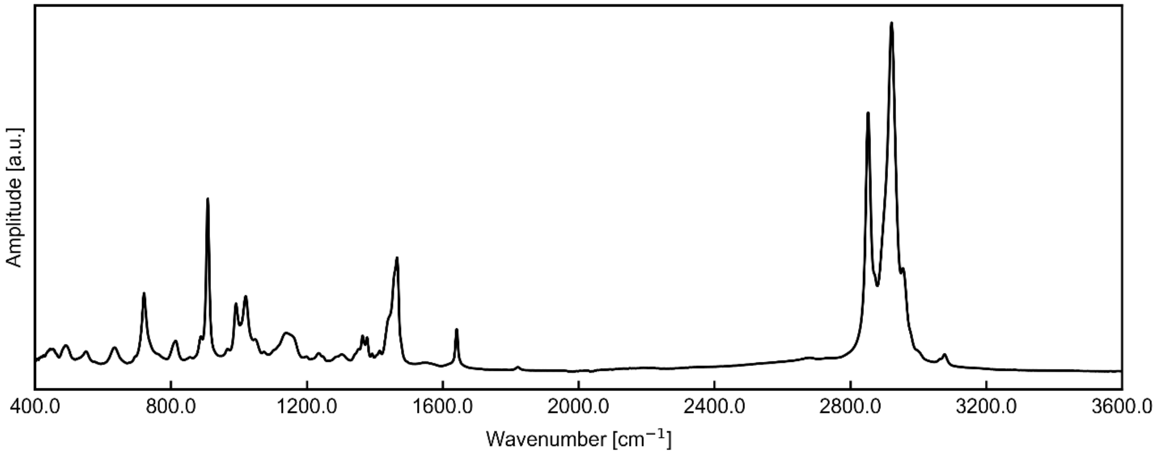

Supplementary Figure 1: FTIR spectrum of the synthetized $CsPbI_3$ nanocrystals in the region 400 - 3600 cm^{-1}.

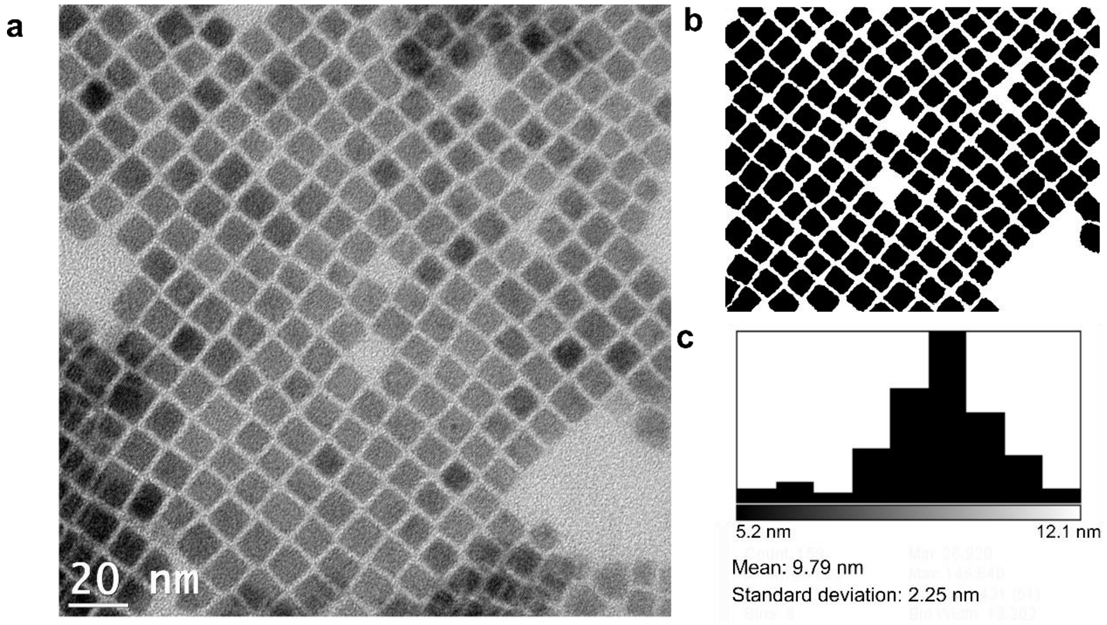

Supplementary Figure 2: a Representative TEM image of the synthetized $CsPbI_3$ nanocrystals. **b** Mask used in image processing software to estimate the size distribution of the nanocrystals. **c** Retrieved histogram from **b**. The mean size of the nanocrystals is 9.8 ± 2.2 nm.

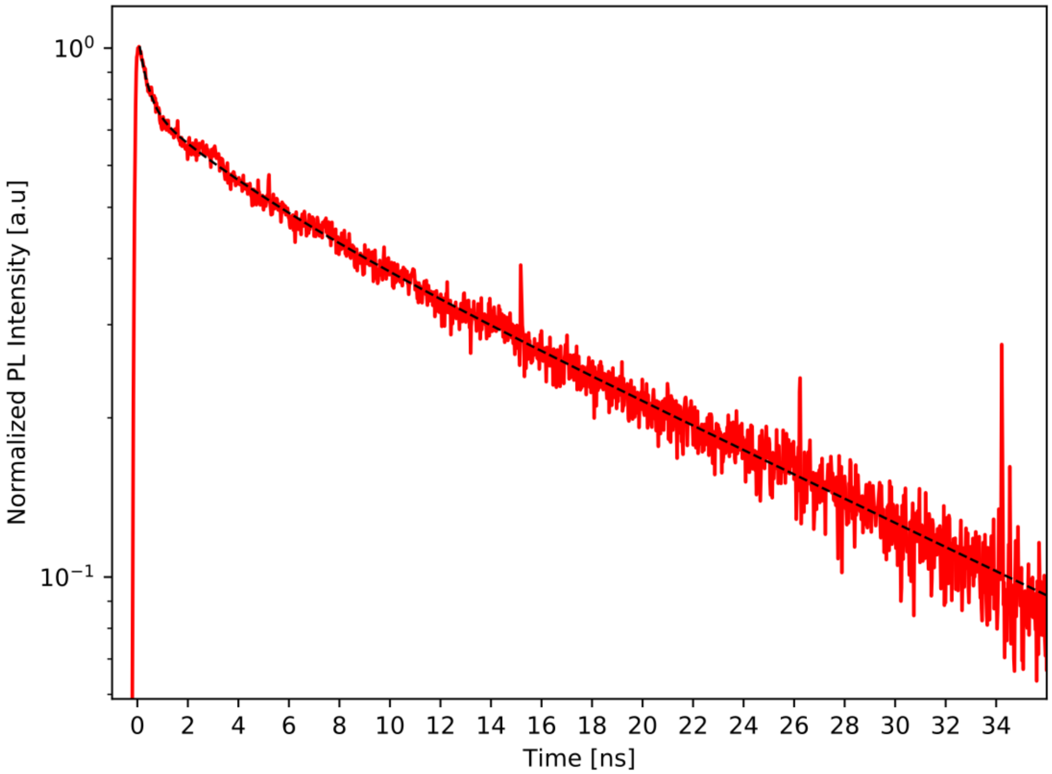

Supplementary Figure 3: Kinetic transient for $CsPbI_3$ at $\langle N \rangle \ll 1$. Summed kinetic transient for $CsPbI_3$ shown in red. Dashed black line shows the fit to a triexponential decay, yielding an average lifetime of 17.25 ns.

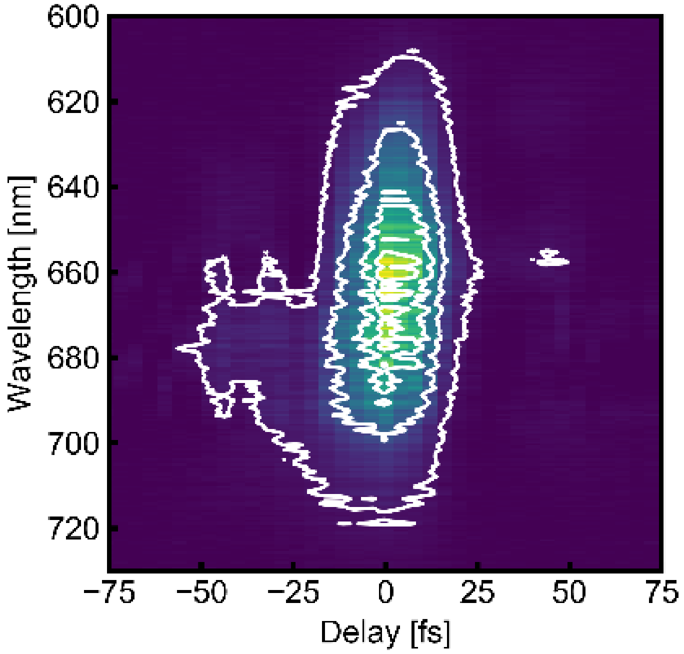

Supplementary Figure 4: TG-FROG trace of the pulses employed in the 2DES experiments on $CsPbI_3$ shown in the main manuscript, corresponding to pulses of 13-15 femtoseconds.

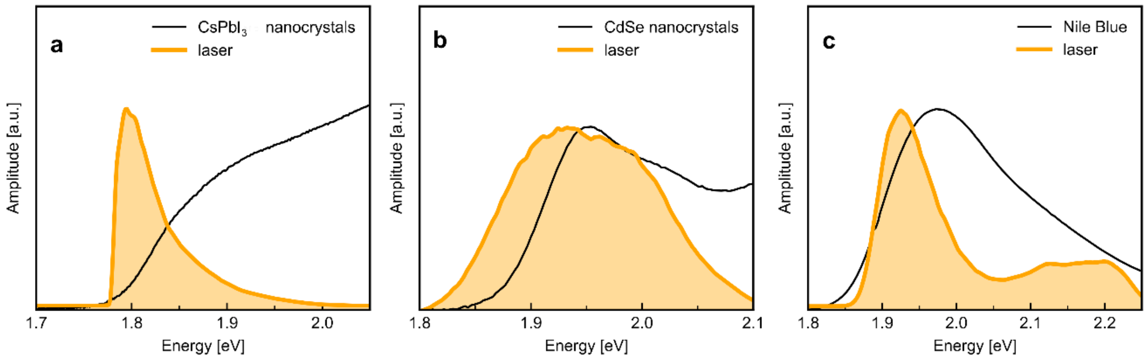

Supplementary Figure 5: Spectral overlap between the laser spectrum and the samples. **a** $CsPbI_3$ nanocrystals in toluene **b** CdSe nanocrystals in toluene **c** Nile Blue in ethanol.

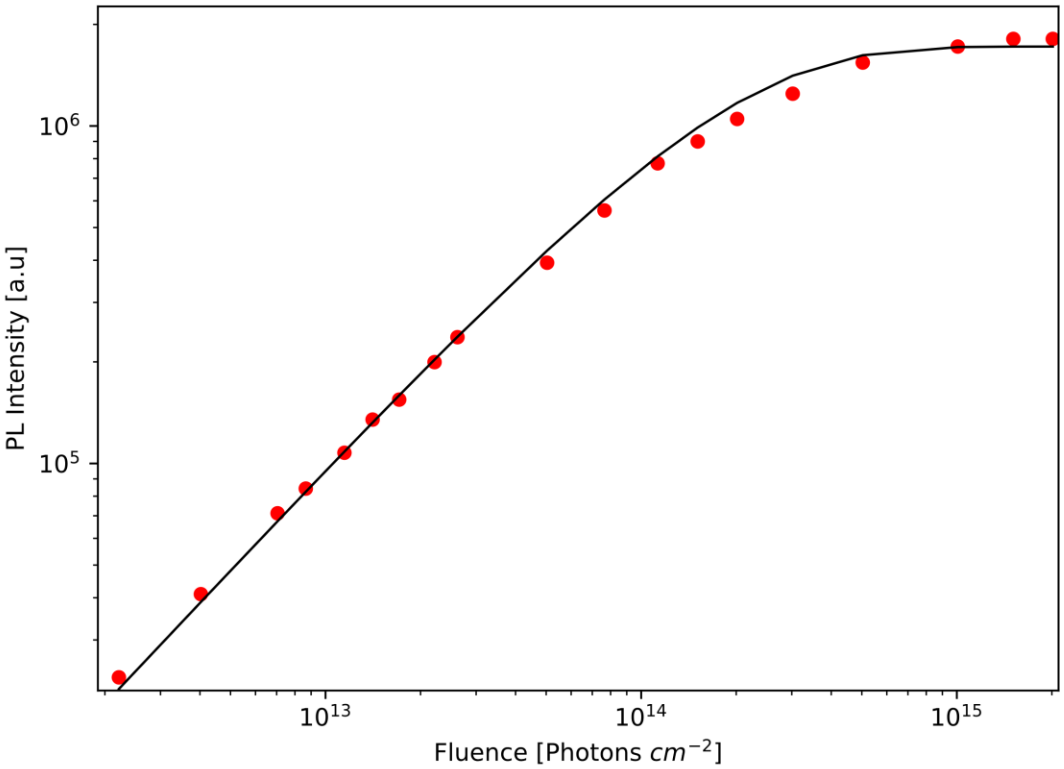

Supplementary Figure 6: Fit of the maximum PL intensity at different fluences as obtained from t-PL data. Red points show the maximum PL intensity after multiexciton decay for a range of fluences. The fit to Poisson statistics is shown by the black line.

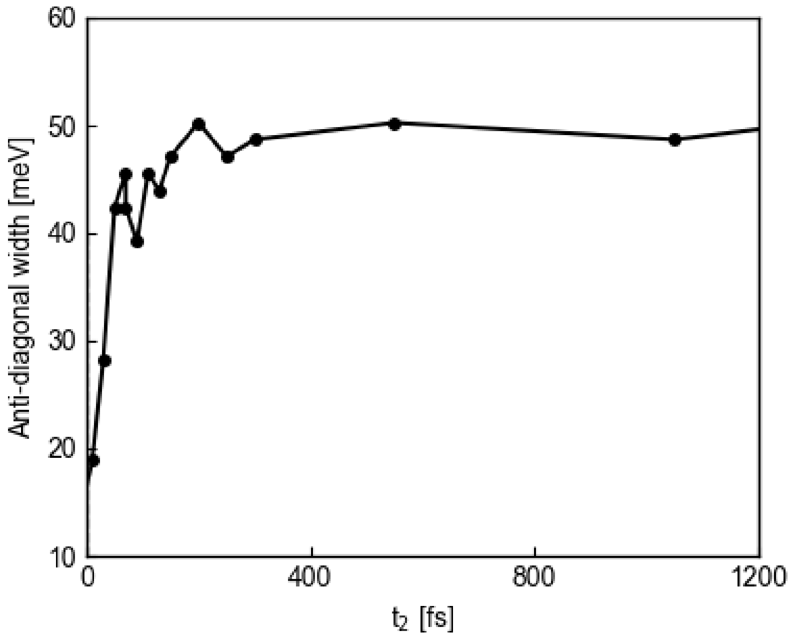

Supplementary Figure 7: The anti-diagonal lineshape behaviour of the perovskite nanocrystals shown in Fig. 3d was reproduced using an independent batch of $CsPbI_3$ nanocrystals.

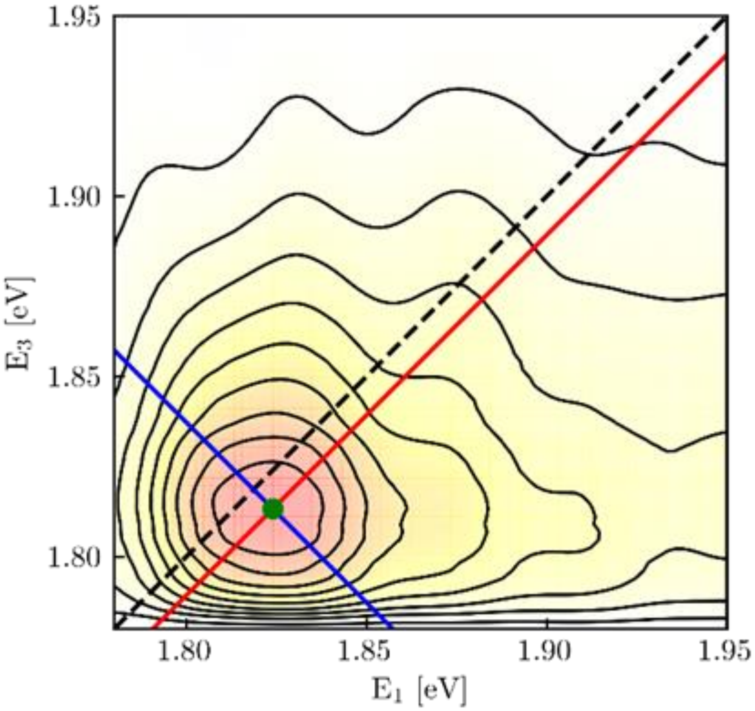

Supplementary Figure 8: Illustration of how the anti-diagonal linewidth (and its FWHM) is extracted from the 2D spectra.

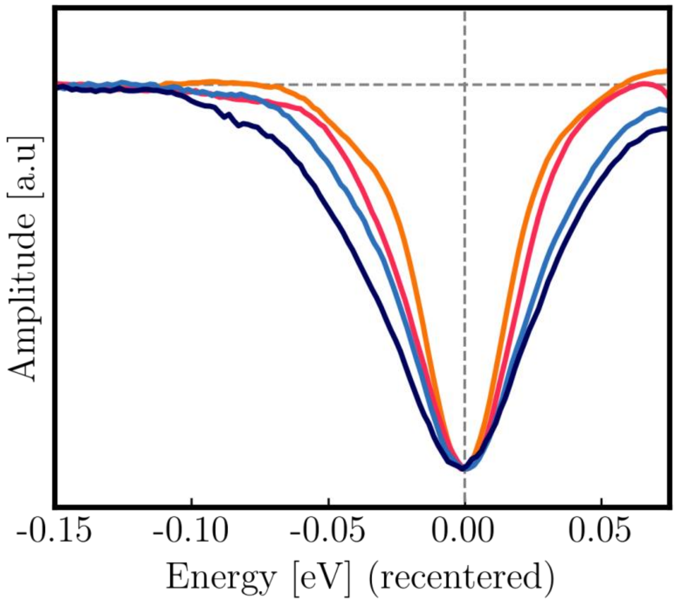

Supplementary Figure 9: Anti-diagonal linewidth (recentered) for exemplary population times. Orange: 10 fs, Red: 30 fs, Light Blue: 100 fs, Dark Blue: 400 fs.

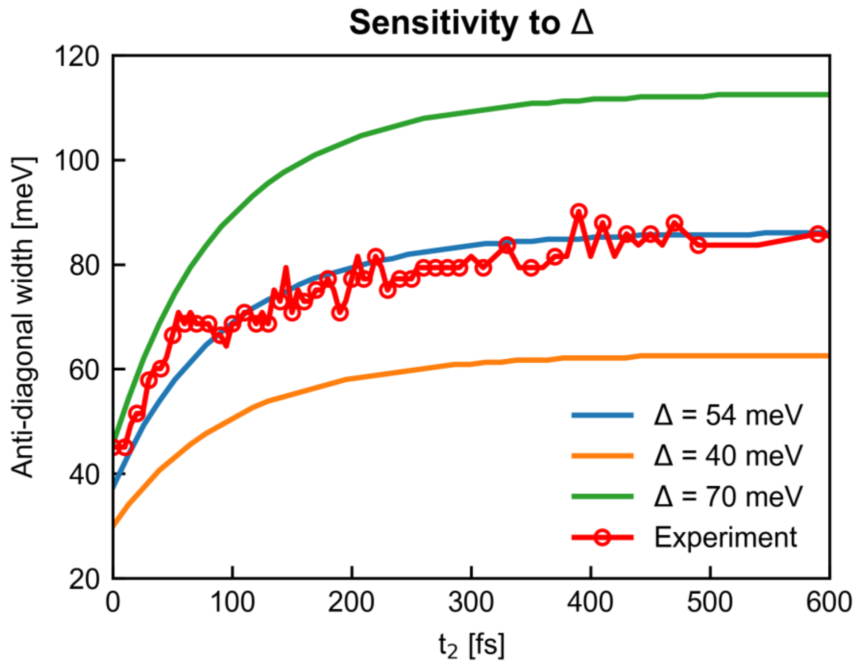

Supplementary Figure 10: Extracted anti-diagonal width from modelled 2D spectra with different values of Δ, showing the sensitivity of the results to 10 meV changes in the value of Δ.

Sample	Pulse energy	Laser spot diameter in sample	OD in 200 μm cuvette	Synthesis
$CsPbI_3$	5 nJ	100 μm	0.3	See "synthesis" section
CdSe	10 nJ	100 μm	0.3	NNlabs
Nile Blue	20 nJ	100 μm	0.3	Sigma Aldrich

Supplementary Table 1: Summary of the experimental parameters for the three samples employed in the study.

Parameter	Value
ω_{01}	1.95 eV
μ_{01}	1
γ	17 meV
σ	25 meV
$S^{(\omega_{\text{vib}})}$	0.15
ω_{vib}	25 meV
T	300 K

Supplementary Table 2: Summary of the experimental parameters employed for the lineshape modelling of CdSe nanocrystals.

Parameter	Value
ω_{01}	1.84 eV
μ_{01}	1
τ_c	110 fs
Δ	54 meV

Supplementary Table 3: Summary of the experimental parameters employed for the lineshape modelling of $CsPbI_3$ nanocrystals.

Supplementary References:

1. J. A. Castañeda, G. Nagamine, E. Yassitepe, L. G. Bonato, O. Voznyy, S. Hoogland, A. F. Nogueira, E. H. Sargent, C. H. B. Cruz, L. A. Padilha, Efficient Biexciton Interaction in Perovskite Quantum Dots Under Weak and Strong Confinement. *ACS Nano*. **10**, 8603–8609 (2016).

2. N. Yarita, H. Tahara, T. Ihara, T. Kawawaki, R. Sato, M. Saruyama, T. Teranishi, Y. Kanemitsu, Dynamics of Charged Excitons and Biexcitons in CsPbBr3Perovskite Nanocrystals Revealed by Femtosecond Transient-Absorption and Single-Dot Luminescence Spectroscopy. *J. Phys. Chem. Lett.* **8**, 1413–1418 (2017).

3. N. S. Makarov, S. Guo, O. Isaienko, W. Liu, I. Robel, V. I. Klimov, Spectral and Dynamical Properties of Single Excitons, Biexcitons, and Trions in Cesium–Lead-Halide Perovskite Quantum Dots. *Nano Lett.* **16**, 2349–2362 (2016).

4. Q. Liu, Y. Wang, N. Sui, Y. Wang, X. Chi, Q. Wang, Y. Chen, W. Ji, L. Zou, H. Zhang, Exciton Relaxation Dynamics in Photo-Excited CsPbI3 Perovskite Nanocrystals. *Sci. Rep.* **6**, 29442 (2016).

5. P. Hamm, M. T. Zanni, *Concepts and methods of 2d infrared spectroscopy* (Cambridge University Press, 2011).

6. B. Li, A. E. Johnson, S. Mukamel, A. B. Myers, The Brownian oscillator model for solvation effects in spontaneous light emission and their relationship to electron transfer. *J. Am. Chem. Soc.* **116**, 11039–11047 (1994).

7. S. Mukamel, D. Abramavicius, Many-body approaches for simulating coherent nonlinear spectroscopies of electronic and vibrational excitons. *Chem. Rev.* **104**, 2073–2098 (2004).

8. H. Seiler, S. Palato, P. Kambhampati, Investigating exciton structure and dynamics in colloidal CdSe quantum dots with two-dimensional electronic spectroscopy. *J. Chem. Phys.* **149**, 074702 (2018).

9. R. Kubo, in *Stochastic Processes in Chemical Physics* (John Wiley & Sons, Ltd, 2006), vol. 15, pp. 101–127.
